# An online Delphi study to investigate the completeness of the CanMEDS Roles and the relevance, formulation, and measurability of their key competencies within eight healthcare disciplines in Flanders

**DOI:** 10.1186/s12909-022-03308-8

**Published:** 2022-04-10

**Authors:** Oona Janssens, Mieke Embo, Martin Valcke, Leen Haerens

**Affiliations:** 1 Department of Educational Studies, Faculty of Psychology and Educational Sciences, Ghent University, H. Dunantlaan 2, 9000 Ghent, Belgium; 2 Department of Movement and Sports Sciences, Faculty of Medicine and Health Sciences, Ghent University, Watersportlaan 2, Ghent, 9000 Belgium; 3 Expertise Network Health and Care, Artevelde University of Applied Sciences, Voetweg 66, Ghent, 9000 Belgium

**Keywords:** Allied health disciplines, CanMEDS, Continuous professional development, Delphi study, Key competencies, Midwifery, Nursing, Roles

## Abstract

**Background:**

Several competency frameworks are being developed to support competency-based education (CBE). In medical education, extensive literature exists about validated competency frameworks for example, the CanMEDS competency framework. In contrast, comparable literature is limited in nursing, midwifery, and allied health disciplines. Therefore, this study aims to investigate (1) the completeness of the CanMEDS Roles, and (2) the relevance, formulation, and measurability of the CanMEDS key competencies in nursing, midwifery, and allied health disciplines. If the competency framework is validated in different educational programs, opportunities to support CBE and interprofessional education/collaboration can be created.

**Methods:**

A three-round online Delphi study was conducted with respectively 42, 37, and 35 experts rating the Roles (*n* = 7) and key competencies (*n* = 27). These experts came from non-university healthcare disciplines in Flanders (Belgium): audiology, dental hygiene, midwifery, nursing, occupational therapy, podiatry, and speech therapy. Experts answered with yes/no (Roles) or on a Likert-type scale (key competencies). Agreement percentages were analyzed quantitatively whereby consensus was attained when 70% or more of the experts scored positively. In round one, experts could also add remarks which were qualitatively analyzed using inductive content analysis.

**Results:**

After round one, there was consensus about the completeness of all the Roles, the relevance of 25, the formulation of 24, and the measurability of eight key competencies. Afterwards, key competencies were clarified or modified based on experts’ remarks by adding context-specific information and acknowledging the developmental aspect of key competencies. After round two, no additional key competencies were validated for the relevance criterion, two additional key competencies were validated for the formulation criterion, and 16 additional key competencies were validated for the measurability criterion. After adding enabling competencies in round three, consensus was reached about the measurability of one additional key competency resulting in the validation of the complete CanMEDS competency framework except for the measurability of two key competencies.

**Conclusions:**

The CanMEDS competency framework can be seen as a grounding for competency-based healthcare education. Future research could build on the findings and focus on validating the enabling competencies in nursing, midwifery, and allied health disciplines possibly improving the measurability of key competencies.

**Supplementary Information:**

The online version contains supplementary material available at 10.1186/s12909-022-03308-8.

## Background

Competency-based education (CBE) constitutes an educational strategy that is widespread in nursing, midwifery, and allied health disciplines (= non-nurse, non-physician healthcare professionals) [1, 2]. It reflects an approach preparing healthcare professionals for practice by defining the competencies students require to meet societal and patient needs. In CBE, objectives are thus reached if competencies are acquired, with the focus lying on the output rather than the input (i.e., accumulation of curriculum hours) [3]. Due to this shift, CBE allows flexibility and learner-centredness [3–5]. Moreover, the shift to CBE might prepare healthcare professionals to work in an interprofessional context as competencies are developed and deployed to support interprofessional education and collaboration [6]. Furthermore, sufficient overlap between competency frameworks of different disciplines might support effective interprofessional collaboration.

Different overarching competency frameworks capturing CBE outcomes have been developed for medical education [3, 7] such as the Canadian Medical Education Directives for Specialists (CanMEDS) [8], the Tomorrow’s Doctors [9], the Scottish Doctor [10], and the Accreditation Council for Graduate Medical Education (ACGME) [11]. Although at the surface, the competencies within each competency framework might differ, the competency descriptions mirror large similarities [3]. The CanMEDS competency framework is organized into seven thematic groups of competencies which are expressed as seven Roles: Medical Expert, Communicator, Collaborator, Leader, Health Advocate, Scholar, and Professional [8]. Within each CanMEDS Role, there are several key competencies. The key competencies refer to the knowledge, skills, and attitudes of a healthcare professional and are described in greater detail by the enabling competencies. The term “enabling competencies” refers to the essential components of a key competency [8]. The authors labelled this framework as a three-level competency framework with “level one”: Roles (e.g., communicator); “level two”: key competencies (e.g., physicians are able to establish professional therapeutic relationships with patients and their families); and “level three”: enabling competencies (e.g., communicate using a patient-centered approach that encourages patient trust and autonomy and is characterized by empathy, respect, and compassion). Slightly different, the ACGME framework starts from six key competency domains, i.e., patient care, medical knowledge, professionalism, interpersonal and communication skills, practice-based learning and improvement, and systems-based practice. The Tomorrow’s Doctors framework refers to the Roles as ‘outcomes’ i.e., ‘the doctor as a scholar and a scientist’, ‘the doctor as a practitioner’, and ‘the doctor as a professional’. The Scottish Doctor framework identifies three outcomes i.e., ‘what the doctor is able to do’, ‘how the doctor approaches his/her practice’, and ‘the doctor as a professional’ [10].

All frameworks build on (future) doctors’ roles, yet some frameworks are more comprehensive than others with CanMEDS and ACGME being the most comprehensive, covering some missing competencies of other competency frameworks as in The Scottish Doctor the collaboration competencies are only present in one single learning outcome and in Tomorrow’s doctors, the Leader Role is lacking [10, 12, 13]. The Leader competencies are also less reflected in the ACGME framework. Nevertheless, the ACGME framework is the only framework putting forward levels of performance by defining milestones to describe competency development [14]. The CanMEDS competency framework might be seen as a comprehensive framework. Only the communication between healthcare professionals, which can be seen as a part of the Collaboration competencies, could be considered as a missing part in CanMEDS, despite the fact there is an emphasis on the Communicator Role (communication with patient and family) and the Collaborator Role (collaboration with colleagues/in team). Building on the above, CanMEDS is seen as the most fitting competency framework for the present study.

In contrast to the extensive literature being available about the use and validation of the CanMEDS competency framework in medical education, comparable literature for nursing, midwifery, and allied health disciplines is limited and a study validating the CanMEDS competency framework including experts from different professions is lacking. The studies on validating discipline-specific competency frameworks (e.g., the essential competencies for midwifery practice from the International Confederation of Midwives [15–19]) regularly relied on the CanMEDS competency framework and integrated the Roles as a guide through the curriculum [20–28]. Despite a large amount of literature confirming the usefulness of the CanMEDS competency framework in medical education and a limited amount of literature describing the validation and use of the CanMEDS competency framework in nursing, midwifery, and allied health disciplines, no studies could be identified evaluating the CanMEDS competency framework involving eight different healthcare disciplines (audiology, dental hygiene, nursing (associate degree and bachelor), midwifery, occupational therapy, podiatry, and speech therapy) [25–27]. Therefore, it remains unclear how to implement the CanMEDS competency framework in nursing, midwifery, and allied health disciplines and if the same CanMEDS competency framework is applicable to different healthcare disciplines [8, 23].

In the current study, we fill this void by examining the CanMEDS competency framework for eight different healthcare disciplines (Copyright© 2015 The Royal College of Physicians and Surgeons of Canada; http://www.royalcollege.ca/rcsite/canmeds/canmeds-framework-e. Reproduced with permission.). The aims of this study are two-fold: (1) to investigate the completeness of the CanMEDS Roles (level 1) and (2) to investigate the relevance, formulation, and measurability of the CanMEDS key competencies (level 2) in eight healthcare disciplines in order to obtain a validated competency framework that might facilitate the implementation of CBE. By simultaneously including experts of eight different educational programs, the current study helps achieving a level of alignment while at the same time respecting the specific nature of each educational program. The ultimate goal is to support healthcare education and especially interprofessional healthcare education and collaboration. Due to our focus on validating an overarching competency framework, we decided not to validate the enabling competencies (level 3). The inclusion of enabling competencies from the start could hinder the applicability of the competency framework in diverse healthcare disciplines and interfere with a focus on interprofessional education and collaboration.

## Methods

A Delphi study was set up based on an online Qualtrics^XM^ survey. The Delphi technique builds on multiple iterations, mostly consisting of two or three rounds, that enable anonymous, systematic refinement of expert opinions to arrive at consensus. Delphi studies have proven to be useful in educational settings to map guidelines, develop curricula, define competencies, etc. [29, 30]. Based on the approach adopted in the study of Michels et al. [25] where the CanMEDS key competencies were examined in a medical education context, the current study evaluated (1) the completeness of the seven CanMEDS Roles (= whether or not each CanMEDS Role was covered by the corresponding key competencies and/or if there were any overlapping key competencies), and (2) their relevance (= relevant enough?), formulation (= clear enough?), and measurability (= assessable enough?) of each of the 27 CanMEDS key competencies in the context of nursing, midwifery, and six allied health disciplines.

### Definition of consensus

No standard definition of the concept ‘consensus’, nor standard threshold values could be found in the literature [31]. Based on a review of the literature about Delphi Studies, consensus in this study was attained when 70% or more of the experts agreed on the question of whether a Role is fully covered by its key competencies (yes/no) (completeness of the Roles). Given the criteria relevance, formulation, and measurability of key competencies, consensus was achieved when 70% or more of experts shared a rating of ≥ 4 on a 6-point Likert-type scale [25]. This value avoids consensus building on the neutral middle point [32–35].

### Forward-translation backward-translation

Since no Dutch translation is available in the literature, the Roles and corresponding key competencies of the CanMEDS framework were first translated into Dutch by five independent researchers, including the main researcher. The original Roles and key competencies were compared to the translated Roles and key competencies [36]. By comparing the translations and taking the context into account, small linguistic adaptations were applied. Next, this Dutch language version was backward-translated by a native English speaker familiar with translating medical educational texts [37] to (1) check the accuracy of the Dutch translation by comparing the backward-translation to the original [36], and (2) to use this English translation to report the study in this paper [36]. In Additional file 1: Appendix 1, an overview of the original key competencies, the forward-translation to Dutch, and the backward-translation to English can be found. During the study, the forward-translated Dutch version was used while the backward-translated English version was used in this paper. Since the validation study did not focus on the enabling competencies, they were not translated.

### Context

This study was conducted in the context of an interdisciplinary research project, aiming at the development of a state-of-the-art ePortfolio tool scaffolding competency development in a large number of healthcare educational programs. Therefore, the study was linked to the following eight healthcare disciplines: audiology, dental hygiene, midwifery, associate degree nursing (EQF level five), bachelor in nursing (EQF level six), occupational therapy, podiatry, and speech therapy [38]. The educational programs are all situated at bachelor level except for the associate nursing degree which is situated at EQF level five in Flanders.

### Sample

A non-probability sampling technique was used by inviting experts from these eight healthcare disciplines employed in 10 educational and seven healthcare institutions in Flanders (not all educational and healthcare institutions were included) [39]. The resulting heterogeneous panel was composed of mentors (healthcare institutions/workplaces), educators (educational institutions/university colleges), educational experts, and board members of hospitals, all familiar with workplace learning. A minimum of three years of expertise in practice was expected to participate to assure that experts had sufficient knowledge about the educational programs they were engaged in [40].

In total, 51 experts were invited to participate in the Delphi study. From these 51, 42 (100%) experts accepted the invitation. In the second round, 37 experts continued their participation (88%), with 35 experts participating in the final round. This drop-out of seven experts from round one until round three (17%) resulted in a final response rate of 83%. Table 1 represents the demographics of the experts. The category ‘other’ within the educational program category clusters all educational experts not affiliated with a specific educational program but involved in work-integrated learning. These experts had an educational background and worked in a hospital or educational institution. They shape and guide healthcare education; e.g., an educational expert involved in different healthcare educational programs in different educational institutions.

**Table 1:** Demographic data of the expert panel (*n* = 42)

GENDER ***n*** = …		AGE (YEARS) ***n*** = …		EDUCATIONAL PROGRAM ***n*** = …		JOB FUNCTION ***n*** = …		EXPERTISE (YEARS) ***n*** = …	
♂	7	20–29	3	NURSING (BACHELOR)	10	EDUCATOR	17	3–5	7
♀	35	30–39	11	NURSING (ASSOCIATE DEGREE)	7	MENTOR	8	5–10	5
		40–49	14	MIDWIFERY	10	EDUCATOR AND MENTOR	2	10–15	15
		50–59	12	SPEECH THERAPY	2	INTERNSHIP COORDINATOR	4	> 15	14
		60–65	2	AUDIOLOGY	4	EDUCATIONAL EXPERT	8	MISSING	1
				OCCUPATIONAL THERAPY	2	BOARD MEMBER	3		
				PODIATRY	2				
				DENTAL HYGIENE	3				
				OTHER	2				

### Data collection

In the current study, a Delphi procedure was set up following three consecutive rounds in which experts were invited to fill out an online survey [41, 42]. To optimize the face and content validity, the survey administration was pilot-tested involving researchers from the research project and an independent content expert [39, 42].

Each of the three rounds lasted two weeks to give experts ample time to complete the survey. The presentation of the CanMEDS framework and the nature of the questions (quantitative/qualitative) changed during the three rounds. The first survey round consisted of a qualitative and a quantitative part. Results from the qualitative part were used to optimize the presentation of the CanMEDS Roles and key competencies in the subsequent round (e.g., offer context-specific formulations, make adjustments, present important concepts, etc.). Round two and three only consisted of a quantitative part. The quantitative parts consisted of scoring the following:

1. **the completeness of the Roles**: the Role is fully covered by its key competencies and there are no missing and/or overlapping key competencies (yes - no).
2. **the relevance, formulation, and measurability of the key competencies:** these criteria were scored on a 6-point Likert-type scale (e.g., very irrelevant - irrelevant – rather irrelevant – rather relevant – relevant - very relevant) [25].

The qualitative part consisted of a text box where experts could add remarks next to each Role and/or key competency.

Table 2 summarizes the focus on the CanMEDS Roles and key competencies within each round.

**Table 2:** Detailed information about the presentation of CanMEDS Roles and key competencies during each Delphi round

CanMEDS Roles	CanMEDS key competencies		
	***Relevance***	***Formulation***	***Measurability***
**Round 1**			
*Quantitative + qualitative*	*Quantitative + qualitative*	*Quantitative + qualitative*	*Quantitative + qualitative*
**Round 2**			
*Quantitative*: only non-validated CanMEDS Roles were presented	*Quantitative:* only non-validated key competencies were presented, enriched with information based on the results of the qualitative analysis in round one	*Quantitative:* only non-validated key competencies were presented, reformulated by adding more context-specific information based on the results of the qualitative analysis in round one	*Quantitative*: only non-validated key competencies were presented with extra information based on the results of the qualitative analysis of round one
**Round 3**			
*Quantitative:* only non-validated CanMEDS Roles were presented	*Quantitative:* only non-validated key competencies were presented, enriched with information based on the results of the qualitative analysis in round one	*Quantitative:* only non-validated key competencies were presented, reformulated by adding more context-specific information based on the results of the qualitative analysis in round one	*Quantitative*: only non-validated key competencies were presented supplemented with the corresponding enabling competencies

### Data-analysis

Agreement percentages were calculated for each round using descriptive statistics in SPSS27©. In addition, the qualitative remarks collected during round one were analyzed with NVivo12©, using inductive content analysis [43]. First, data were read multiple times to get immersed in the data. Next, the organizing process started including open coding, creating categories, and abstraction.

Between subsequent rounds, about two weeks were used for data-analysis and preparation of the next round. The duration of these periods was kept as short as possible to reduce drop-out [44]. When analysis revealed consensus was reached for the criterion ‘completeness’ of a Role (≥70% scored ‘yes’), this Role was no longer considered in the next round. When analysis revealed that consensus was reached for relevance, formulation, or measurability (≥70% rated ≥4 on the 6-point Likert-type scale), the key competency was no longer presented in the consecutive round. For the formulation criterion, only the modified key competencies were taken to the next round.

## Results

### Completeness of the Roles (level 1)

**Fig. 1:**
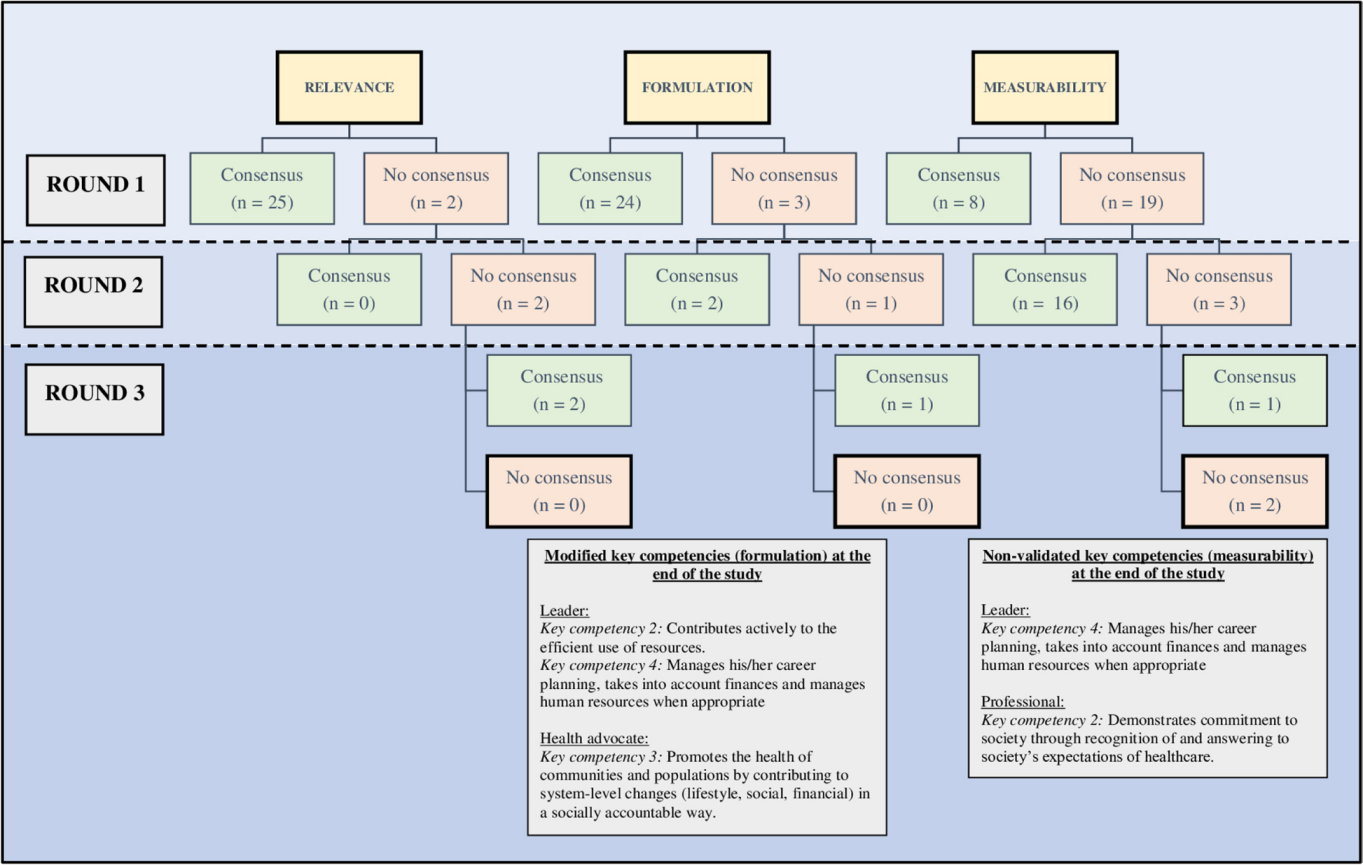
Flow chart of the validation process

The analysis of the data of round one revealed that all Roles (*n* = 7) were fully covered by their corresponding key competencies and that no overlapping key competencies were identified. Agreement between experts is shown in the following percentages: Expert (92%), Communicator (92%), Collaborator (92%), Leader (92%), Health Advocate (92%), Scholar (84%), and Professional (87%). Below in Fig. 1, a flow chart of the validation process can be found reporting the number of key competencies with/without consensus for relevance, formulation, and measurability on the occasion of each round.

### Relevance, formulation, and measurability of the key competencies (level 2)

The evaluation of the relevance, formulation, and measurability of the key competencies consisted of two parts: a qualitative part and a quantitative part.

### Qualitative analysis

**Fig. 2:**
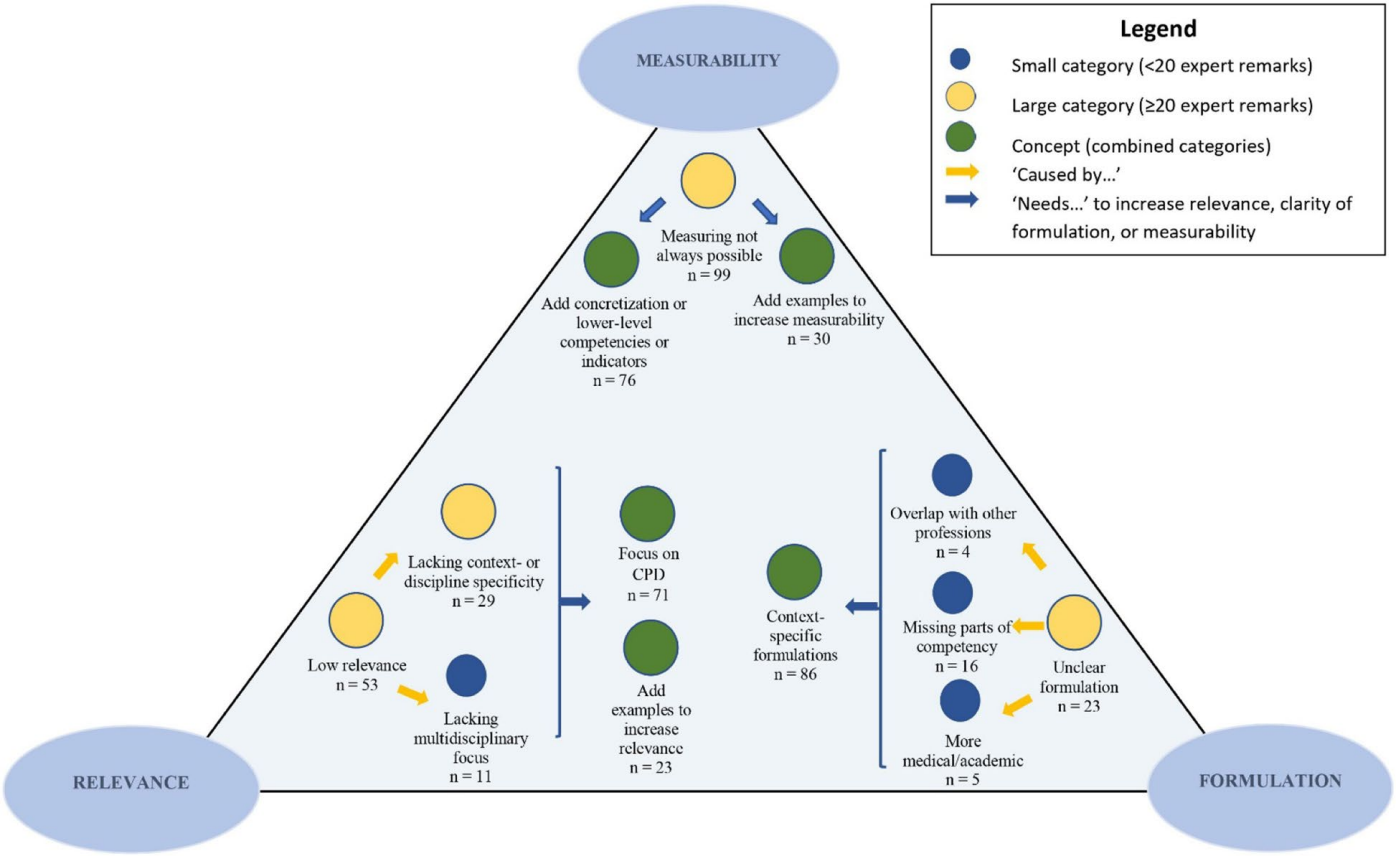
Visualization of the qualitative analysis of experts' remarks

Below, Fig. 2 gives a visualization of the qualitative analysis of the remarks of experts within the first round. First, expert remarks were coded. Second, these codes were aggregated into categories. The number of expert remarks per category was displayed in Fig. 2 (*n* = …). The blue circles represent small categories (*n* = 4) reflected in less than 20 expert remarks. The yellow circles represent large categories (*n* = 4) with input from at least 20 expert remarks. After forming categories, the abstraction phase started analyzing the links between the categories by forming concepts. The green circles represent concepts (*n* = 5). These concepts were further analyzed and were used as a base to formulate recommendations in view of the subsequent validation process (*n* = 1) (context-specific formulations), or in view of the future implementation process of the competency framework (*n* = 4) (necessary concretization or concrete competencies/indicators, examples to increase measurability, Continuous Professional Development (CPD), and examples to increase relevance). The arrows refer to links between the categories/concepts. A complete overview of codes (*n* = 208), small categories (*n* = 4), large categories (*n* = 4), and concepts (*n* = 5) can be found in Additional file 1: Appendix 2.

### Quantitative analysis

Table 3 gives an overview of the quantitative analysis results. The percentages are reported for each key competency after each round (R1-R3).

**Table 3:** Quantitative analysis process

ROLES	KEY COMPETENCIES (Backward-translation)	RELEVANCE			FORMULATION			MEASURABILITY		
*EXPERT*	*A healthcare professional…*	*R1*	*R2*	*R3*	*R1*	*R2*	*R3*	*R1*	*R2*	*R3*^*b*^
	Operates within own occupational remit and ability.	97.6%	–	–	90.2%	–	–	82.9%	–	–
	Carries out clinical assessment and implements a patient-centric care plan.	100%	–	–	95.1%	–	–	68.3%	100%	–
	Plans and carries out investigations and “therapies” for the purpose of diagnosis and/or treatment.	95.1%	–	–	82.9%	–	–	63.4%	91.4%	–
	Puts in place a care plan for continuation of treatment, and further consultation as necessary.	97.6%	–	–	78%	–	–	63.4%	100%	–
	Actively contributes to the continued improvement of healthcare quality and patient safety, both as an individual, and as part of a team.	100%	–	–	95.1%	–	–	61%	82.2%	–
*COMMUNICATOR*	Builds professional (therapeutic) relationships with clients/patients and their families.	94.7%	–	–	94.7%	–	–	78.9%	–	–
	Accurately collects and synthesizes relevant information, taking into account the perspectives of clients/patients and their families.	100%	–	–	92.1%	–	–	84.2%	–	–
	Shares information about health conditions and plans with clients/patients and their families.	100%	–	–	92.1%	–	–	71%	–	–
	Involves clients/patients in the development of the care plan that reflect the needs and goals of the patient.	100%	–	–	86.8%	–	–	63.2%	94.3%	–
	Documents and shares written and electronic contact information about the client / patient to optimize the clinical diagnosis, client/patient safety, confidence and privacy.	97.4%	–	–	78.9%	–	–	81.6%	–	–
*COLLABORATOR*	Works purposefully with other care givers in both own and other disciplines.	94.7%	–	–	92.1%	–	–	70.3%	–	–
	Works with colleagues from own and other disciplines to foster understanding, acknowledge differences and resolve conflict.	94.7%	–	–	91.1%	–	–	60.5%	85.2%	–
	Transfers the care of client/patient to other physicians, facilitating continuity of care.	94.7%	–	–	100%	–	–	60.5%	97.1%	–
*LEADER*	Contributes to the improvement of healthcare in teams, organizations and systems.	92.1%	–	–	84.2%	–	–	47.4%	83.3%	–
	Engages in the management of healthcare resources. **Contributes actively to the efficient use of resources**^**a**^**.**	73.7%	–	–	52.6%	86.1%	–	29%	75%	–
	Demonstrate leadership in professional practice.	86.8%	–	–	86.8%	–	–	57.9%	83.3%	–
	Manages career planning, finance, and human resources of the practice. **Manages his/her career planning, takes into account finances and manages human resources when appropriate**^**a**^**.**	60.5%	57.6%	74.3%	60.5%	80.6%	–	18.4%	63.9%	62.9%
*HEALTH ADVOCATE*	Addresses the individual needs of the client/patient by discussing his/her health inside and outside the clinical environment.	100%	–	–	86.8%	–	–	65.8%	85.5%	–
	Addresses the health needs of communities or populations by lobbying for systemic changes in a socially responsible manner. **Promotes the health of communities and populations by contributing to system-level changes (lifestyle, social, financial) in a socially accountable way**^**a**^**.**	68.4%	66.7%	74.4%	60.5%	58.3%	88.5%	13.2%	48.6%	71,4%
*SCHOLAR*	Commits to the continuing improvement of professional activities through lifelong learning.	100%	–	–	100%	–	–	60.5%	94.5%	–
	Educates students and teaching physicians, colleagues in other disciplines and the wider population.	92.1%	–	–	94.7%	–	–	44.7%	88.9%	–
	Introduces best available evidence-based insights into practice.	97.4%	–	–	94.7%	–	–	68.4%	94.5%	–
	Contributes to the creation and dissemination of knowledge and new practices applicable to health.	89.5%	–	–	84.2%	–	–	34.2%	91.7%	–
*PROFESSIONAL*	Demonstrates commitment to clients/patients through the application of best practice and ethical standards.	100%	–	–	92.1%	–	–	86.8%	–	–
	Demonstrates commitment to society through recognition of and answering to society’s expectations of healthcare.	92.1%	–	–	73.7%	–	–	31.6%	52.8%	68.6%
	Demonstrates commitment to the profession through the adherence to professional standards and corresponding legal requirements (law).	97.4%	–	–	92.1%	–	–	78.9%	–	–
	Demonstrates commitment to personal health and wellbeing in order to promote optimal care.	92.1%	–	–	76.3%	–	–	52.6%	69.5%	–

Extra information

^a^Validated context-specific formulations

^b^In round 3, enabling competencies were added to validate the measurability criterion

After the first round, consensus was attained about the relevance of 25 out of 27 key competencies. Key competencies reflecting no consensus were related to (1) the Leader Role: manages career planning, finance, and human resources of the practice; and (2) the Health Advocate Role: addresses the health needs of communities or populations by lobbying for systemic changes in a socially responsible manner.

The qualitative analysis illustrated how the relevance of not yet validated key competencies could be increased by making context-specific adjustments or adding clarifying examples (e.g., ‘this key competency could be measured through the performance of a task or simulation’). After presenting recommendations in round two, this did still not result in consensus about the relevance of both key competencies. In round three, the importance of these key competencies to be reached as part of CPD was emphasized instead of during the bachelor’s or associate’s degree. This resulted in consensus for both key competencies. This implies that experts consider these key competencies to be attained after graduation.

For the formulation, consensus was reached for 24 out of 27 key competencies after round one. No consensus was reflected about the following key competencies: (1) engages in the management of healthcare resources (Leader), (2) manages career planning, finance, and human resources of the practice (Leader), and (3) addresses the health needs of communities or populations by lobbying for systemic changes in a socially responsible manner (Health Advocate).

Qualitative analysis revealed that context-specific formulations were necessary to enhance formulation. These context-specific formulations were constructed based on experts’ remarks. This resulted in achieving consensus about two more key competencies after round two. In round three, after rephrasing, consensus was also achieved for the last key competency. Final formulations of the three modified key competencies are documented in bold in Table 3.

After round one, no consensus was achieved about the measurability of 19 out of the 27 key competencies. Qualitative analysis pointed at the need for concretization and adding examples. Furthermore, some key competencies were identified to be attainable, and thus measurable, only after graduation. Furthermore, experts suggested that ePortfolios might be effective tools to measure or monitor competency development. In round two, experts could now judge whether key competencies were measurable by the end of the educational program or after graduation. Experts reached consensus for 24 out of the 27 key competencies. Problems were identified about the following key competencies: (1) manages career planning, finance, and human resources of the practice (Leader), (2) addresses the health needs of communities or populations by lobbying for systemic changes in a socially responsible manner (Health Advocate), and (3) demonstrates commitment to society through recognition of and answering to society’s expectations of healthcare (Professional). In round three, corresponding enabling competencies were added for these three key competencies, based on the qualitative analysis, to make non-validated key competencies more concrete. As the validation of the enabling competencies was not our primary goal the enabling competencies were not translated to Dutch through robust backward-forward procedures. We therefore presented the original English enabling competencies to the experts to concretize and validate the key competencies. This resulted in the validation of an additional key competency within the Health Advocate Role. No consensus could be reached for the following two key competencies: (1) manages career planning, finance, and human resources of the practice (Leader), and (2) demonstrates commitment to society through recognition of and answering to society’s expectations of healthcare (Professional).

## Discussion

Literature and practice support the idea that validated competency frameworks can guide CBE. In the medical education field, literature is available about a variety of competency frameworks that have been developed and validated. This is not the case for nursing, midwifery, and the six allied healthcare educational programs. Therefore, this Delphi study is the first study investigating the CanMEDS competency framework in view of its adoption in nursing, midwifery, as well as six allied healthcare educational programs. The aim of this study was to evaluate (1) the completeness of the Roles (level 1), and to investigate (2) the relevance, formulation, and measurability of the key competencies (level 2). The results of three consecutive Delphi rounds show consensus about the completeness of the Roles after round one. The relevance and formulation of the key competencies were validated after round three. Twenty-seven key competencies were validated after round three for the measurability criterion. Two key competencies are yet not validated in terms of the measurability criterion: (1) manages career planning, finance, and human resources of the practice (Leader), and (2) demonstrates commitment to society through recognition of and answering to society’s expectations of healthcare (Professional). Several concepts emerged from the data. These concepts were used to influence consecutive rounds and might also be seen as important results for future research. The concepts that emerged from the data related to the relevance criterion were: (1) focus on CPD and (2) add examples to increase the relevance. The concept that supports the formulation criterion was: form context-specific formulations. The concepts for the measurability criterion were: (1) add concretization or lower-level competency indicators, and (2) add examples to increase measurability. The following discussion mainly builds on these concepts (Fig. 2).

### Concretization by enabling competencies (level 3)

The validation of an overarching competency framework creates opportunities to support interprofessional learning and collaboration due to the achieved uniformity [16]. As such the primary goal of this study was to validate an overarching competency framework that can drive CBE in eight different healthcare educational programs and not to provide an assessment tool nor a list with assessable behavioral indicators. Therefore, it was decided not to validate the enabling competencies. Yet, the measurability of the higher-level Roles (level 1) and key competencies (level 2) was poorly rated during the first two Delphi rounds. As such, we ultimately decided to add the enabling competencies (level 3) to clarify three key competencies in Delphi round three. After adding the enabling competencies, experts indeed rated one additional key competency as measurable. Literature shows that assessment indeed is the most challenging process when implementing a competency framework as competencies rather describe professional behavior than observable and measurable actions [7]. Van der Lee et al. [45] confirm this statement, stressing how abstract and general descriptions of the Roles and key competencies provide a clear and relevant framework but emphasizing how underlying enabling competencies are needed to measure and assess these in educational settings. Compared to our findings, in one of the comparable studies set up in the medical education field, Michels et al. [25] also emphasized the necessity of making Roles and key competencies better measurable and concrete. Our findings confirm that adding enabling competencies could be a solution to improve measurability without losing the interprofessional opportunities of the higher-level Roles and key competencies if there are adapted to a specific healthcare educational program.

### Context-specificity and discipline-specificity

Two key competencies could not be validated in terms of the measurability criterion: (1) manages career planning, finance, and human resources of the practice (Leader), and (2) demonstrates commitment to society through recognition of and answering to society’s expectations of healthcare (Professional). The issue remained also after adding enabling competencies or adding a CPD angle. The issues can be partly explained by the observation that these key competencies might fall outside the context of nursing, midwifery, and allied healthcare education and rather be geared to the medical context as reflected in the original CanMEDS competency framework. The question remains whether nursing, midwifery, and allied healthcare educational programs miss out on these competencies or whether they are indeed less crucial in view of these programs. There are options to deal with the two non-validated key competencies. As we already emphasized the role of CPD, some healthcare educational programs might opt to shift these competencies forward in the educational continuum and to prioritize them at a later stage. This does not imply we consider these competences as less essential for nursing, midwifery, and allied healthcare education. An alternative option is to screen current healthcare educational programs and to check whether these two key competencies represent a weak or blind spot in current curriculum design being addressed in future research. Van der Lee et al. [45] and Dent et al. [46] recommend the addition of context-specificity to the key competencies. As our qualitative analysis confirms these findings, the measurability of the non-validated key competencies could be improved by offering enabling competencies – as stated earlier - but appropriate in the context of each involved expert rather than offering generic enabling competencies appropriate for a medical context.

### The developmental aspect of competencies

Although our results showed that the CanMEDS Roles seem to be covered by the related key competencies, the validation process pointed out some questions remain about some Roles when looking through a healthcare educational lens. The Leader Role did seem difficult to validate as there was no consensus about the relevance of one key competency and the measurability of all four key competencies when a linkage with CPD was not added (in round two). Also, key competencies related to the Professional Role seemed difficult to validate in nursing, midwifery, and the six allied healthcare educational programs. Herion et al. [47] found that the Leader Role was perceived as less relevant than other Roles, even after graduation. Apparently, experts in our study rather considered these Roles and corresponding key competencies as relevant for future professional life and less essential in the context of an educational program, especially linked to their measurability. Furthermore, competency frameworks different from CanMEDS also put less emphasis on the Leader Role. For instance, in Tomorrow’s Doctors, the Leader Role is not included [12]. The latter framework was also developed for a bachelor’s degree level – though medical – students. This suggests that key competencies related to this Role might be targeted in master degree healthcare professionals, implying that key competencies related to the Leader Role are not geared to bachelor’s or associate’s degree healthcare educational programs but could be linked to CPD. Furthermore, these findings might be in accordance with the vision of Edgar et al. [48] who stated that competency frameworks are not developed to support short internships, but rather to support longer rotations emphasizing the importance of continuing education.

Given the important place of CPD in developing competencies, the developmental aspect of competencies needs to be considered, not only before but also after graduation. The CanMEDS competency framework presents ‘milestones’ reflecting the expected development of competencies during the educational program, when transitioning to practice, and also during practice. Although these milestones reflect an emphasis on competency growth, they are insufficiently detailed to evaluate the actual development of competencies during an educational program [6]. The ACGME framework offers more detail in describing performance levels (novice, advanced beginner, competent, proficient, and resident/expert), but also these levels are insufficient to guide a developmental assessment of the competencies [7]. Also, taxonomies, such as Miller’s pyramid [49] and Dreyfus’ levels in skills acquisition [50] put forward levels but lack detail in only providing a vague description of expected performance levels. The above reflects a finding shared in the medical education literature when looking at the implementation potential of current tools [51]. This could be tackled by supplementing abstract performance levels with detailed and concrete indicators of expected behavior. The latter could be done at the level of the enabling competencies that are enriched with expected behavioral outcomes in specific contexts.

### Interprofessional education and collaboration

The Collaborator Role in the CanMEDS competency framework lacks a focus on interprofessional communication competencies. Although communication is crucial to collaborate effectively within a team [23], experts did not comment on these missing interprofessional competencies. Some reasons can be assumed such as: (1) experts were not yet familiar with the CanMEDS competency framework and focused on the presented competencies rather than on the missing competencies, or (2) interprofessional communication was seen as belonging to the Communicator Role. Future research will focus on expanding the Collaborator Role by developing and validating specific interprofessional communication competencies.

### Recommendations for practice

Building on our results, the following checklist could be used to support the implementation of the CanMEDS competency framework in nursing, midwifery, and six allied healthcare educational programs:

- □ The CanMEDS competency framework is complete, relevant, and clear to support CBE in nursing, midwifery, and several allied healthcare educational programs.
- □ Consider implementing the CanMEDS competency framework at the level of key competencies to allow different healthcare educational programs to implement a shared competency framework. The implementation of a shared framework offers opportunities for interprofessional education and collaboration. To take into account the specific nature of an educational program, different educational programs could put forward their own enabling competencies and add these to the shared framework. In this way, key competencies are less vague, more workable, and better measurable and assessable.
- □ Consider capturing growth during the educational program as some key competencies cannot be fully developed at the beginning of a program. Including a focus on different educational levels (from an associate degree to a master’s degree) might allow to map growth along the entire educational continuum. The same applies when focusing on CPD to capture competency growth after graduation [52, 53]. The CanMEDS competency framework does provide a Competence Continuum where the transition to a master’s degree and the transition to practice are visualized. This could serve as a base to visualize competency growth before and after graduation in an ePortfolio context [8].

## Future research

Our findings point at the value of the CanMEDS competency framework to support CBE in nursing, midwifery, and six allied healthcare educational programs. This opens an avenue for future research to examine how this competency framework can be implemented. Our findings show the relevance of zooming in on the three levels, being the Roles, key competencies, and enabling competencies, which is in accordance with a study of Michels et al. [25]. An interesting track for future research might be to investigate why the yet non-validated key competencies are difficult to measure in these healthcare disciplines. Accordingly, although enabling competencies were added in round three to make non-validated key competencies more concrete, future research might build on this study to focus on more systematically validating the complete CanMEDS enabling competencies, possibly adapted to a specific context in healthcare education. Moreover, adding levels of performance might increase the relevance, as well as the measurability of key competencies and enabling competencies, supporting the educational continuum [48].

As measuring key competencies remains difficult, capturing the key competencies and competency growth in an ePortfolio, as suggested by the experts, offers new opportunities. Most ePortfolios (e.g., PebblePad, Mahara, etc.) serve as learning spaces where students store and document their work in line with competencies and related indicators; where they reflect on their learning trajectory; where they assess a collection of their work; and that help them to showcase their accomplishments [54]. This pushes forward CBE since these functionalities are related to reflection, feedback, assessment, and showcasing evidence; both by students as by educators or mentors [55]. Moreover, ePortfolios – for the predefined competencies – push students to link their practical experiences to the backbone of their program. By collecting and documenting evidence derived from practical experiences, ePortfolios help capturing the educational continuum. Step by step, student enrich in the ePortfolio the evidence base in terms of measurable outcomes [28]. When ePortfolios build on a shared competency framework in different educational programs – such as CanMEDS adopted in the current study - this opens avenues for interprofessional education and collaboration. Students and staff from different educational programs will be able to work together and engage in peer-feedback or joint planning of patient care during internships [56]. Lastly, a design-based research study design can be adopted to examine how particular ePortfolio design features, help students reach predefined competencies including interprofessional competencies resulting in better patient care [57].

## Limitations

First, a convenience sample was used. Therefore, there is a chance that the sample might not be representative for the entire population. To minimize sampling bias, we included experts from different healthcare educational programs and different healthcare and educational institutions. Furthermore, the sample size was large enough to allow for including a heterogeneous sample. Another limitation might be related to the small number of experts within some healthcare disciplines (audiology: *n* = 4; dental hygiene: *n* = 3; occupational therapy: *n* = 2; podiatry: *n* = 2; speech therapy: *n* = 2). Some of these educational programs (audiology, dental hygiene, and podiatry) are relatively new and the actual size of these educational programs is small compared to the established field of nursing and midwifery.

The decision not to validate the enabling competencies might be seen as a limitation. Nevertheless, the validation of the Roles and key competencies helped attaining an overarching, shared competency framework for different healthcare disciplines. This already increases the opportunities for interprofessional education and collaboration. The developmental phases of the CanMEDS competency framework included in the Competence By Design (CBD) initiative were not taken into consideration because our aim was to validate the Roles and key competencies within seven bachelor degree and one associate degree healthcare educational program. Nevertheless, this represents an interesting strand of future research..

There are other healthcare educational programs where the CanMEDS competency framework has been used e.g., pharmacy, dentistry, and physical education, specialist nurses (e.g., nurse anesthetists). These educational programs were not included in our study. Nevertheless, the modified CanMEDS competency framework can be the starting point for educators from other healthcare disciplines aiming at contextualizing the CanMEDS competency framework to their discipline [23, 47, 58–60].

## Conclusions

This study investigated whether the CanMEDS Roles and key competencies offer a base to develop a uniform competency framework to support CBE in nursing, midwifery, and several allied healthcare educational programs. The results of this study help conclude that CanMEDS is a valuable base to give direction to healthcare education. The validated – and slightly adapted - competency framework presented in the current study is a starting point to develop CBE for nursing, midwifery, and six allied healthcare educational programs. But the results also suggest the potential to give direction to interprofessional education and collaboration, and CPD. Hopefully, the study findings will inspire healthcare educational programs to grab the opportunity to standardize their competency frameworks across different educational institutions and programs to support CBE.

## Supplementary Information

**Additional file 1: Appendix 1.** Original, forward-translated, and backward-translated CanMEDS key competencies. **Appendix 2.** Overview of concepts, categories, and codes.

## Data Availability

A Data Management Plan was constructed via https://dmponline.be and monitors the storage and access to the data (ID: 107491). Data are available on request by contacting the main author (Oona.Janssens@UGent.be).

## Abbreviations

CPD: Continuous Professional Development
CBE: Competency-based education
CanMEDS: Canadian Medical Education Directives for Specialists
ACGME: Accreditation Council for Graduate Medical Education
ICM: International Confederation of Midwives
