# Supplementary material for: An online Delphi study to investigate the completeness of the CanMEDS Roles and the relevance, formulation, and measurability of their key competencies within eight healthcare disciplines in Flanders
Source: BMC Med Educ. 2022 Apr 10;22:260. doi: 10.1186/s12909-022-03308-8 (PMC8994879; doi:10.1186/s12909-022-03308-8)
Supplement: Supplementary file 1 — Additional file 1: Appendix 1. Original, forward-translated, and backward-translated CanMEDS key competencies. Appendix 2. Overview of concepts, categories, and codes. [file 12909_2022_3308_MOESM1_ESM.docx]

# Supplementary Info

# Appendix 1: original, forward-translated, and backward-translated CanMEDS key competencies

| **Original CanMEDS key competencies**  **(the physician is able to…)** | **Forward-translation to Dutch**  **(de zorgverlener…)** | **Backward-translation to English**  **(the healthcare professional…)** |
| --- | --- | --- |
| **EXPERT** | | |
| Practise medicine within their defined scope of practice and expertise. | Handelt binnen de eigen beroepsbevoegdheid en -bekwaamheid. | Operates within own occupational remit and ability. |
| Perform a patient-centred clinical assessment and establish a management plan. | Voert een (klinische) beoordeling uit en stelt een plan van aanpak op waarbij de cliënt/patiënt centraal staat. | Carries out clinical assessment and implements a patient centric care plan. |
| Plan and perform procedures and therapies for the purpose of assessment and/or management. | De zorgverlener plant en voert onderzoeken en therapieën uit met beoordeling en/of behandeling als doel. | Plans and carries out investigations and “therapies” for the purpose of diagnosis and/or treatment. |
| Establish plans for ongoing care and, when appropriate, timely consultation. | De zorgverlener stelt een zorgplan op ifv continuïteit van zorg, en wanneer nodig, tijdige consultatie. | Puts in place a care plan for continuation of treatment, and further consultation as necessary. |
| Actively contribute, as an individual and as a member of a team providing care, to the continuous improvement of health care quality and patient safety. | De zorgverlener draagt, als individu en teamlid binnen de zorgverlening, actief bij aan de continue verbetering van de kwaliteit van de gezondheidszorg en de patiëntveiligheid. | Actively contributes to the continued improvement of health care quality and patient safety, both as an individual, and as part of a team. |
| **COMMUNICATOR** | | |
| Establish professional therapeutic relationships with patients and their families. | Bouwt professionele (therapeutische) relaties op met cliënten/patiënten en hun families. | Builds professional (therapeutic) relationships with clients/patients and their families. |
| Elicit and synthesize accurate and relevant information, incorporating the perspectives of patients and their families. | Verzamelt en synthetiseert accurate en relevante informatie, rekening houdend met de perspectieven van cliënten/ patiënten en hun families. | Accurately collects and synthesizes relevant information, taking into account the perspectives of clients/patients and their families. |
| Share health care information and plans with patients and their families | Deelt gezondheidszorginformatie en plannen met cliënten/patiënten en hun families. | Shares information about health conditions and plans with clients/patients and their families. |
| Engage patients and their families in developing plans that reflect the patient’s health care needs and goals | Betrekt cliënten/patiënten en hun families in de ontwikkeling van plannen die de gezondheidszorgnoden en -doelen van cliënten/patiënten weergeven. | Involves clients/patients in the development of the care plan that reflect the needs and goals of the patient. |
| Document and share written and electronic information about the medical encounter to optimize clinical decision-making, patient safety, confidentiality, and privacy | Documenteert en deelt geschreven en elektronische informatie over het cliënt/patiënt contact om de klinische besluitvorming, cliënt/patiënt veiligheid, vertrouwelijkheid en privacy te optimaliseren. | Documents and shares written and electronic contact information about the client / patient to optimise the clinical diagnosis, client/patient safety , confidence and privacy. |
| **COLLABORATOR** | | |
| Work effectively with physicians and other colleagues in the health care professions. | Werkt doeltreffend samen met zorgverleners van eigen en andere disciplines. | Works purposefully with other care givers in both own and other disciplines. |
| Work with physicians and other colleagues in the health care professions to promote understanding, manage differences, and resolve conflicts. | Werkt met zorgverleners van eigen en andere disciplines samen om wederzijds begrip te bevorderen, om te gaan met verschillen en conflicten op te lossen. | Works with colleagues from own and other disciplines to foster understanding, acknowledge differences and resolve conflict. |
| Hand over the care of a patient to another health care professional to facilitate continuity of safe patient care. | Draagt de zorg van een cliënt/patiënt over aan een andere zorgverlener om continuïteit van veilige zorg te faciliteren. | Transfers the care of client/patient to other physicians, facilitating continuity of care. |
| **LEADER** | | |
| Contribute to the improvement of health care delivery in teams, organizations, and systems. | Draagt bij aan de verbetering van zorgverlening in teams, organisaties en systemen. | Contributes to the improvement of healthcare in teams, organizations and systems. |
| Engage in the stewardship of health care resources. | Engageert zich voor het beheer van gezondheidszorgmiddelen. | Engages in the management of healthcare resources. |
| Demonstrate leadership in professional practice. | Toont leiderschap in de professionele praktijkvoering. | Demonstrate leadership in professional practice. |
| Manage career planning, finances, and health human resources in a practice. | Beheert carrièreplanning, financiën en human resources in de praktijkvoering. | Manages career planning, finance and human resources of the practice. |
| **HEALTH ADVOCATE** | | |
| Respond to an individual patient’s health needs by advocating with the patient within and beyond the clinical environment | Beantwoordt aan individuele noden van de cliënt/patiënt door samen over zijn/haar gezondheid te overleggen binnen en buiten de klinische omgeving. | Addresses the individual needs of the client/patient by discussing his/her health inside and outside the clinical environment. |
| Respond to the needs of the communities or populations they serve by advocating with them for system-level change in a socially accountable manner | Beantwoordt aan noden van gemeenschappen of bevolkingsgroepen door het samen pleiten voor een verandering op systeemniveau op een sociaal verantwoorde manier. | Addresses the health needs of communities or populations by lobbying for systemic changes in a socially responsible manner. |
| **SCHOLAR** | | |
| Engage in the continuous enhancement of their professional activities through ongoing learning. | Engageert zich tot continue verbetering van de professionele activiteiten door levenslang te leren. | Commits to the continuing improvement of professional activities through lifelong learning. |
| Teach students, residents, the public, and other health care professionals. | Onderwijst studenten, zorgverleners in opleiding, collega's van andere disciplines en de bevolking. | Educates students and teaching physicians, colleagues in other disciplines and the wider population. |
| Integrate best available evidence into practice. | Integreert best beschikbare evidence-based inzichten in de praktijk. | Introduces best available evidence-based insights into practice. |
| Contribute to the creation and dissemination of knowledge and practices applicable to health. | Draagt bij tot de creatie en verspreiding van kennis en praktijkvoering toepasbaar op de gezondheid. | Contributes to the creation and dissemination of knowledge and new practices applicable to health. |
| **PROFESSIONAL** | | |
| Demonstrate a commitment to patients by applying best practices and adhering to high ethical standards. | Toont toewijding aan cliënten/patiënten door toepassing van beste praktijkvoering en ethische standaarden. | Demonstrates commitment to clients/patients through the application of best practice and ethical standards. |
| Demonstrate a commitment to society by recognizing and responding to societal expectations in health care. | Toont toewijding aan de maatschappij door het herkennen van, én het beantwoorden aan maatschappelijke verwachtingen in de gezondheidszorg. | Demonstrates commitment to society through recognition of and answering to society’s expectations of healthcare. |
| Demonstrate a commitment to the profession by adhering to standards and participating in physician-led regulation. | Toont toewijding aan het beroep door toepassing van standaarden en beroeps-specifieke wetgeving. | Demonstrates commitment to the profession through the adherence to professional standards and corresponding legal requirements (law). |
| Demonstrate a commitment to physician health and well-being to foster optimal patient care. | Toont toewijding aan de persoonlijke gezondheid en welzijn om optimale zorg te bevorderen. | Demonstrates commitment to personal health and wellbeing in order to promote optimal care. |

# Appendix 2: overview of concepts, categories, and codes

| **Concepts** | **Categories (fat) –** Codes | Codes |
| --- | --- | --- |
| **Alternative formulations** |  |  |
|  | To plan and to conduct are different things |  |
|  | Difference between showing and taking leadership |  |
|  |  |  |
|  | **Proposal wording** |  |
|  |  | Reference to patient rights |
|  |  | as an individual and team member make a long sentence |
|  |  | within healthcare instead of on health |
|  |  | add contextualization |
|  |  | the cyclic process from diagnosis to evaluation comes up little |
|  |  | to share is a confusing wording: electronic, oral, or in consultation |
|  |  | sharing is contradictory with privacy and confidentiality |
|  |  | attributes to the expansion and dissemination of knowledge |
|  |  | transfers inter- and interprofessional care of a patient |
|  |  | using efficiently instead of managing |
|  |  | disconnect own and other disciplines |
|  |  | To engage is not clear |
|  |  | only within the clinical environment |
|  |  | there is a missing word before ‘timely consult’ |
|  |  | timely consult is unclear |
|  |  | evaluation and adjustment of intervention is missing |
|  |  | evaluates treatments in order to improve quality of care |
|  |  | expertise or advice as alternative formulations |
|  |  | make family singular |
|  |  | no ‘when necessary’ |
|  |  | healthcare resources in unclear |
|  |  | name boundaries of competencies |
|  |  | the word for evaluation is not used in Flanders |
|  |  | with environment WHEN NECESSARY |
|  |  | add the international aspect |
|  |  | clinical environment is unclear |
|  |  | link between care plan and consultation is unclear |
|  |  | social expectations is vague |
|  |  | management of practice is missing |
|  |  | takes leadership instead of shows leadership |
|  |  | not only to improve, but also to grant afterwards |
|  |  | teach is unclear |
|  |  | examination or podiatric dd instead of treatment |
|  |  | especially head nurse, staff, etc. |
|  |  | system level and in a socially responsible way is unclear |
|  |  | planning and performing AFTER care plan |
|  |  | practice management is vague |
|  |  | RELEVANT data |
|  |  | respect instead of understanding |
|  |  | strives for the best possible quality of care |
|  |  | to large; split up |
|  |  | management is unclear and not fitting the context |
|  |  | replace therapeutic by well-being-enhancing |
|  |  | timely follow-up instead of timely consultation |
|  |  | commitment is vague |
|  |  | shows CLINICAL leadership in |
|  |  | shows entrepreneurship |
|  |  | leaving the word ‘and’, double competency |
|  |  | care plan instead of action plan |
|  |  | takes care of personal health instead of commitment to personal health |
| **Authorities of student** |  |  |
|  | evaluation by student, action plan not |  |
|  | staying within professional boundaries is important |  |
| **Better fit to other role** |  |  |
|  | better within Professional Role |  |
|  | LLL more within Professional Role than Scholar |  |
|  | also within Collaborator Role |  |
| **Competency not clear** |  |  |
|  | complex wording |  |
|  | attributes can be formulated more actively |  |
|  | wording unclear whether it’s about Q of care of interprofessional collaboration |  |
|  | difficult wording |  |
|  | unclear who’s personal health it is about |  |
|  | not clear for everyone |  |
|  | unclear competency |  |
|  | shared decision making is unclear |  |
|  | role of the perspective of the patient is unclear |  |
|  |  |  |
|  | **confusion with first competency** |  |
|  |  | add collaboration in for the sake of the patient to avoid overlap with next competency |
| **Concretising communication competencies** |  |  |
|  | better naming of the communication as a condition for collaboration |  |
|  | communication mostly not with family |  |
|  | communication needs to be aligned with the patient |  |
|  | communication has to be adjusted to persons and contexts |  |
|  | context is important |  |
|  | professional relationship is necessary |  |
|  | consent of patient is needed |  |
| **Content of competency is complex in practice** |  |  |
|  | is not always happening after graduation |  |
|  | very complex if other cultures, religions, etc. |  |
| **Context or discipline specific** |  |  |
|  | within associate degree nursing is this difficult |  |
|  | context-specificity is necessary |  |
|  | only for nursing |  |
|  | within associate degree nursing, they have to check their expertise with higher educated persons |  |
|  | difficult to measure within associate degree educational programs |  |
|  | less relevant for associate degree nursing |  |
|  | role of expert less relevant within associate degree nursing |  |
|  | very present within associate degree nursing |  |
|  | especially masters, head nurses, staff, independent healthcare professional, coordinator, etc. |  |
| **Continuing Education** |  |  |
|  | continuing education - before graduation |  |
|  | continuing education_longer internships are necessary |  |
|  | CPD_especially after graduation |  |
|  | expected level is necessary |  |
|  | expectations differ between programs |  |
| **Examples to allow or increase measurability of competencies** |  |  |
|  | can be measured by working economically or ecologically with material |  |
|  | by reflection and evaluation |  |
|  | by evaluation of the training profile |  |
|  | by observation, motivation or accountability |  |
|  | by translation professional knowledge to non-professionals |  |
|  | in function of continuity of care |  |
|  | by reflections |  |
|  | by internship assignments |  |
|  | giving information to other students |  |
|  | taking knowledge from classes to workplace |  |
|  | by patient satisfaction surveys |  |
|  | by asking questions and checking whether the student knows what he/she is doing |  |
|  | by ePortfolios |  |
| **Examples to clarify relevance of competencies** |  |  |
|  | life-long learning attitude is important |  |
|  | error management |  |
|  | planning is mostly the transfer to the mentor |  |
|  | proactive action |  |
|  | student needs insight for that |  |
|  | student can re-evaluate, propose another plan and thinking critically about the purposefulness of the therapy |  |
|  | encourage the team to reflection and self-directness is also important within leadership |  |
|  | propagate patient safety |  |
|  | mostly focus on students |  |
|  | perform what they have learned |  |
|  | good transfer can fit here_necessary for continuity |  |
|  | assertiveness and conflict management |  |
|  | within the course organization and legislation |  |
|  | ethical conflict between professional and social expectations |  |
|  | health information and education cycle_motivating conversations |  |
|  | in the context of research |  |
|  | in the context of data transfer |  |
|  | network care |  |
|  | also the well-being of others and adjusting culture |  |
|  | refers to anamnesis |  |
|  | trainings, educating a student, etc. |  |
|  | development of time-bound projects |  |
| **Individual level – micro** |  |  |
|  | on an individual level, less as a team member |  |
|  | especially on a micro level, less on a macro level |  |
|  | especially on a micro level, macro is depending on the context |  |
| **Interdisciplinarity - multidisciplinarity necessary** |  |  |
|  | transfer to dentist |  |
|  | IPEC competencies could form a framework for the collaborator role |  |
|  | this is no individual responsibility |  |
|  | emphasis more on team-player than leadership |  |
|  | consultation of multidisciplinary team is necessary |  |
|  | collaboration with general practitioner is necessary |  |
|  | especially interprofessional communication |  |
| **Interfaces-overlap with other professions** |  |  |
|  | overlap with medical professions |  |
|  | overlap with other healthcare professions |  |
| **Low relevance** |  |  |
|  | does not belong to task package |  |
|  | only within first consultation-introduction to a patient |  |
|  | not students’ qualifications |  |
|  | not a goal itself |  |
|  | leadership is not always present |  |
|  | less relevant-focus on individual |  |
|  | not always or everywhere relevant |  |
|  | collaboration with own discipline is rare |  |
|  | insufficiently present in practice |  |
|  | to observe but not to conduct as a student |  |
| **Measuring-assessing not always possible** |  |  |
|  | qualification difficult to measure because of multiple educational structured e.g., associate degree nurse vs. bachelor nurse |  |
|  | only one-time measurement possible |  |
|  | workplace is necessary-patient has to be followed-up from beginning until end |  |
|  | measurement of evaluation by mentor |  |
|  | measurement only possible in team meeting |  |
|  | difficult to measure |  |
|  | opportunities to measure competencies |  |
|  | opportunities to measure competencies\measurable at the level of taking initiative |  |
|  | opportunities to measure competencies\improving measurability by alternative internship concepts |  |
|  | opportunities to measure competencies\measuring possible by electronic patient file |  |
|  | measuring not always and everywhere possible |  |
|  | can not always be observed_motivation is necessary |  |
|  | especially last-year students |  |
| **Necessary concretization of competencies** |  |  |
|  | Concretization within competency is necessary |  |
|  | Too vague_concretization is necessary |  |
| **Parts of competencies are missing** |  |  |
|  | understandable communication is missing |  |
|  | treatment but also diagnosis |  |
|  | digital aspect is missing within Collaborator Role |  |
|  | evaluating treatment is missing within Expert Role |  |
|  | focus on intramural care is missing |  |
|  | acting within the framework of professional deontology is important within the Professional Role |  |
|  | international aspect is missing within the Collaborator Role |  |
|  | clinical reasoning is missing as a competency within the Expert Role |  |
|  | critical or methodical thinking and acting are missing within the Scholar Role |  |
|  | critical thinking and self-reflection are missing |  |
|  | quality of care could be added within the Leader Role |  |
|  | life-long learning is missing within the Professional Role |  |
|  | next to evidence-based also practice-based |  |
|  | technical skills are missing |  |
|  | the use of technology is missing within the Communicator Role |  |
| **Recognition of clarity** |  |  |
| **Recognition of measurability** |  |  |
| **Recognition of relevance** |  |  |
|  | difficult to determine within non-chronic care |  |
| **Relevance and measurability depending on internship and context** |  |  |
|  | depending on the internship context |  |
|  | not always possible to act independently |  |
| **Role more medical-academic** |  |  |
|  | Role of Leader sounds weird in healthcare |  |
|  | Role of Scholar is very academic |  |
|  | Too medical |  |
|  | Too medical\no nursing jargon |  |
